# Editorial: Exploring personal recovery and development in forensic mental health

**DOI:** 10.3389/fpsyt.2023.1194439

**Published:** 2023-05-04

**Authors:** Henning Hachtel, Birgit Angela Völlm

**Affiliations:** ^1^Department of Forensics, University Psychiatric Clinic Basel, Basel, Switzerland; ^2^Department of Forensic Psychiatry, University Hospital Rostock, Rostock, Mecklenburg-Vorpommern, Germany

**Keywords:** recovery, development orientation, criminal recidivism, forensic care, implementation, hindrances

The challenge to implement and validate the benefits of recovery-oriented interventions and attitudes in secure settings or court-mandated treatments remains. In this Research Topic, the scientific and clinical community contributed to studies regarding different aspects of recovery-related outcomes, implementation, and comparisons with other models of psychiatric treatments.

Firstly, the published literature on this topic adds to existing data, which hints at the beneficial prospect of recovery-oriented interventions in forensic mental health, regarding the overall goal of criminal recidivism and symptom improvement (Hofmann et al.; Lutz et al.; Rees and Thomson). This contributes to the ongoing discussion on whether recovery-oriented interventions should be established.

Secondly, at an individual level of the person being mandated to treatment in forensic settings, the improvement of self-awareness and identity (Kovács et al.), as well as self-efficacy (Schoppmann et al.) are manifold; furthermore, positive changes have been demonstrated in personal wellbeing as well as an influence on possible criminogenic factors (Lutz et al.; Rees and Thomson). Empowerment seems to be connected to a possible rise in motivation and taking responsibility for the client. The support of family members regarding recovery pathways of persons with severe mental disorders is a widely recognized impact factor. Barriers to taking an active part in the care of these persons for family members outside of feelings of guilt or shame are discussed in the study by Rowaert et al.. The family's need for support for themselves during the care trajectory is often overlooked.

Hindrances and difficulties in implementing a recovery orientation and the accompanying problems of cultural change of attitudes in staff and organizations are to be taken into consideration. Uncertainty of staff, structural and security obstacles, as well as underlying themes of losing control are to be expected (Schoppmann et al.). Clinical management should address and be aware of these key points. The benefits of implementing recovery-oriented approaches in form of peer support are described by clients and broaden the therapeutic possibilities of engagement and support (Walde et al.). While implementing recovery orientation in forensic mental health settings (e.g., clinics) seems to be worth considering, detained people in prisons, who are suffering from a high burden of mental health problems, should not be left out of these considerations (Weber et al.).

The challenge of recovery orientation goes hand in hand with embracing a new clinical philosophy, promoting service user participation in their care and treatment. While services should meet the needs of men and women who are mentally ill or have a personality disorder, there are profound implications in forensic mental health settings (1). But if reducing the risk of reoffending is promoted by helping service users build more meaningful lives and getting more intensely involved in therapeutic activities by strengthening their participation, the effort is well-justified for the client, the institution, and society.

## Author contributions

HH wrote the first draft of the manuscript and contributed to conception and design of the Research Topic. Both authors contributed to manuscript revision, read, and approved the submitted version.
